# Changing men or changing health systems? A scoping review of interventions, services and programmes targeting men’s health in sub-Saharan Africa

**DOI:** 10.1186/s12939-021-01428-z

**Published:** 2021-03-31

**Authors:** Thierry Beia, Karina Kielmann, Karin Diaconu

**Affiliations:** 1 Health Services Department, Copperbelt University, Jambo Drive, Riverside, Kitwe, Zambia; 2 Institute for Global Health and Development, Queen Margaret University, Edinburgh, Musselburgh, EH21 6UU Scotland, UK

**Keywords:** Men’s health, Sub-Sahara Africa, Health literacy, Health seeking

## Abstract

**Background:**

Sex and gender have been shown to influence health literacy, health seeking behaviour, and health outcomes. However, research examining the links between gender and health has mainly focused on women’s health, which is a long-standing global health priority. We examine literature focused on the ‘missing men’ in global health research, in particular empirical studies that document interventions, programmes, and services targeting men’s health issues in Sub-Saharan Africa. Within these studies, we identify dominant conceptualisations of men and men’s health and how these have influenced the design of men’s health interventions and services.

**Methods:**

This is a scoping review of published and grey literature. Following comprehensive searches, we included 56 studies in the review. We conducted a bibliographic analysis of all studies and used inductive methods to analyse textual excerpts referring to conceptualizations of men and service design. An existing framework to categorise services, interventions, or programs according to their gender-responsiveness was adapted and used for the latter analysis.

**Results:**

From the included studies, we distinguished four principal ways in which men were conceptualized in programs and interventions: men are variously depicted as ‘gatekeepers’; ‘masculine’ men, ‘marginal’ men and as ‘clients. Additionally, we classified the gender-responsiveness of interventions, services or programmes described in the studies within the following categories: gender-neutral, −partnering, −sensitive and -transformative. Interventions described are predominantly gender-neutral or gender-partnering, with limited data available on transformative interventions. Health systems design features – focused mainly on achieving women’s access to, and uptake of services – may contribute to the latter gap leading to poor access and engagement of men with health services.

**Conclusion:**

This review highlights the need for transformation in sub-Saharan African health systems towards greater consideration of men’s health issues and health-seeking patterns.

**Supplementary Information:**

The online version contains supplementary material available at 10.1186/s12939-021-01428-z.

## Background

The inter-related dynamics of sex and gender are acknowledged to be important influences on health literacy, health seeking, and health outcomes [1, 2]. In global health and development, research on gender and health has historically focused on women’s health [3]. Emphasis on women’s health has both ideological roots in the feminist and women’s health movements as well as practical immediacy in the recognition of women’s sex- and gender-specific risks and vulnerabilities.

More recently, there has been growing interest in men’s health issues and an appeal to include men in discussions of gender and health, and gender-sensitive health programming [4, 5]. Launched in March 2018, the Global Health 50/50 report appeals for global health organizations to redress the ‘inconvenient truths’ of men’s health gaps, and the absence of attention to gender dynamics in global heath agenda-setting [6]. In many parts of the world, men engage less with health services than women, are less likely to access preventive services, and are more likely to drop out of care [4, 7–11]. Although well-documented in high income countries [12], this phenomenon is becoming a focus of research in low- and middle-income countries (LMIC), and can be partly explained by two trends in global health programming. First, the Cairo Population and Development conference in 1994 [13] allowed a focus on sexual and reproductive health (SRH) to emerge as a distinct ‘stream’ of donor funding and programmatic focus, leading to more concerted efforts to examine ways in which the SRH needs of women throughout the life cycle could be met through both stand-alone and integrated service delivery models. Within this context, it became evident that for women living in many countries, men’s involvement was important in enabling program effectiveness and impact in the areas of family planning, abortion, pregnancy care and maternal health [14]. Initiatives to strengthen men’s engagement with SRH programs have therefore largely targeted and regarded men as partners or prospective fathers [15, 16].

Second, throughout the 2000s, the growth of global health initiatives, targeted funding, and vertical programming for control of infectious diseases such as HIV, tuberculosis (TB), and malaria, as well as neglected tropical diseases led to exponential growth in operational research on access, uptake and utilization of services related to these programs. Donor-driven program targets and improved information systems led to documentation of the ‘missing men’ in three areas: access to diagnostic and treatment services, poor uptake of services, and poor retention in care. Research on HIV and TB care in Southern Africa has indicated that men are consistently more likely to disengage with health services than women and have higher mortality for HIV and TB [17–20]. These gaps in men’s health are likely to persist and worsen with the rise of noncommunicable diseases (NCD) and complex patterns of co-morbidity.

Examining how researchers and health planners frame and conceptualise men and male health-seeking behaviours is a critical first step to understanding the gaps in design, organisation, and implementation of men’s health services. To this purpose, we conducted a scoping review of peer-reviewed and grey literature that (a) describe or evaluate interventions or programmes that have been adopted to improve men’s health literacy and health-seeking behaviour and/or (b) document men’s experience with accessing health services in sub-Saharan Africa. Our regional focus is justified given the relative paucity of data in this region despite increased interest in male engagement in SRH programming. We paid particular attention to how men and men’s health-related behaviours are conceptualized and explore how these conceptualizations relate to the types of interventions deployed to address men’s health literacy and health seeking. We adapted the framework put forward by Gupta and colleagues [21] to classify interventions, services and programs discussed across the literature, and further, to identify factors associated with increased health literacy and health seeking. The study provides a topical overview of the research conducted to date in the region and identifies opportunities and directions for future development of interventions to strengthen men’s health literacy and health-seeking towards better health outcomes.

## Methods

We conducted a scoping review following Arksey and O’Malley’s methodological framework [22] as updated by Levac et al. [23] and the Joanna Briggs Institute [24]; this is an established methodology for appraising the state of a field of research [25]. We discuss the operationalization of each scoping review stage below, with the exception of the optional stage of stakeholder consultation [22], which we did not conduct. The scoping review forms part of a larger study that explores perspectives of both global experts as well as key informants (KI) in a Southern African setting; triangulation of findings from the KI study and this review will be reported on separately.

### Stage 1: Specifying objectives and research questions

The aim of the review was to examine current research and literature on intervention approaches to strengthening men’s health literacy and health-seeking behaviour in Sub-Saharan Africa.

Specifically, in relation to empirical studies undertaken in Sub-Saharan Africa, we addressed the following research questions:

1. What is the aim, scope of activity and coverage of studies documenting interventions, programs and services that target men’s health literacy and health-seeking?
2. What conceptualizations of men underlie the interventions, programs and/or services described in literature and how do these conceptualizations relate to intervention, program, or service design as presented?
3. For the interventions, programs and/or services described, what factors are identified by authors as being linked to increased health literacy and health seeking?

### Stage 2: Identifying relevant studies

The following electronic databases were searched in May 2019: MEDLINE, CINAHL, PsychInfo, and Web of Science, OpenGrey and Grey Literature Report were also searched to identify relevant grey literature. A sample search strategy as conducted in Medline is provided in Appendix 1. To ensure all relevant studies were located, we additionally further manually searched through the references of included studies.

### Stage 3: Study selection

We included articles published since 2000 considering that the past 20-year period corresponds to the gradual rise of global interest in the influence of gender in health, and more specifically the gaps in reporting on men’s health issues, specifically in the arena of global health [4, 26, 27]. Studies conducted in English, French, and Portuguese, with men aged 15 years and above, reporting on health services, interventions, or programmes, focusing on health literacy or health seeking were selected for inclusion. We focused on studies conducted in Sub-Sahara Africa (SSA)—understood as the region of Africa located to the south of the Sahara Desert and constituted of 47 countries [28]. These countries can be grouped into four main sub regions, namely Southern Africa, Eastern Africa, Central Africa, and Western Africa. A detailed list of eligibility criteria is provided in Appendix 2.

Two reviewers independently and sequentially screened the titles, abstracts, and then full texts of retrieved publications. The first reviewer conducted an initial screening of all retrieved references to provide a quality check and ensure that criteria were sufficiently well specified to capture documents of interest. The second reviewer then reviewed a random 20% of studies and compared their screening outcome against that of the first reviewer. A Kappa of 75% was pre-agreed as a necessary condition for screening studies in this way; if agreement between reviewers was below this, all studies were to be double screened. Comparison of screening suggested an agreement in inclusion/exclusion of studies of above 85%.

### Stage 4: Extraction and charting data

In line with research questions, a data extraction form was developed and progressively refined in consultation with the research team (see Appendix 3). Extracted information included: bibliometric study identifiers, study objectives, designs and methods, conceptualization of men, description of the intervention, programme or service, and theory of change (ToC) and study findings with respect to men. If no ToC was explicitly stated, we extracted relevant quotes referring to underlying assumptions and/or desired pathways of change. The data extraction process was piloted by two reviewers using a sample of five included studies. Inconsistencies and disagreements were addressed through discussion until a consensus was reached. All data was extracted into Excel 2016; summary tables and charts of the data were prepared via this software as well.

### Stage 5. Synthesis of the results

Overarching trends across studies were summarized via bibliometric analyses. Narrative synthesis was used to summarize data on conceptualizations of men, on theories of change underlying interventions, and on study findings with respect to men [22]. For data on conceptualizations of men and their health seeking behaviour and literacy, we inductively analysed extracted quotes and data. The research group identified and critically discussed patterns in the data and any divergent cases. Further, we iteratively derived a typology of how men were conceptualised in the studies reviewed.

For data on the theories of change and assumptions underlying interventions, we adapted the conceptual framework proposed by Gupta and colleagues [21] to categorise services, interventions or programs according to their gender-responsiveness, that is, the extent to which gender-specific issues were taken into account in the planning, execution and evaluation of health services/interventions. Initially conceived for HIV programmes, the framework by Gupta and colleagues was adopted by the WHO as a model to analyse the extent of integration of gender issues and challenges into programmes and policy making. In our adaptation, we distinguished gender sensitive-partnering from other gender-sensitive interventions given the high levels of SRH programming in the Sub-Saharan African region. We thus expanded the scope of application of the framework by classifying services, interventions, or programs against one of four categories of gender-responsiveness including: neutral, sensitive, partnering, and transformative.

### Reporting

We reported findings according to the PRISMA guideline for scoping reviews [29], however, we chose not to perform the optional quality assessment as this was not necessary in relation to the questions guiding the review.

## Results

**Fig. 1:**
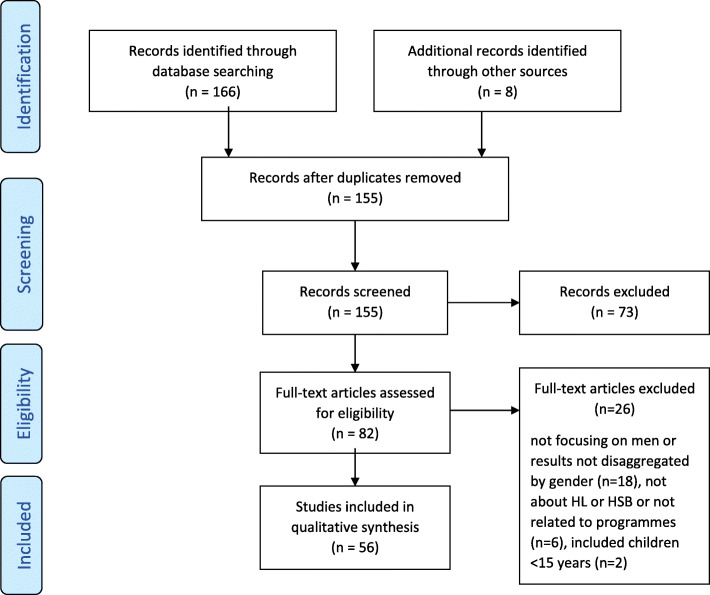
Study flow diagram

Figure 1 shows details of the study selection process. The electronic database search identified 166 studies. Eight more studies were retrieved through manual search and the snowballing process. After removing duplicates, 155 titles and abstracts were screened and studies that did not meet the inclusion criteria were removed.

Full texts of 82 studies were assessed for eligibility: 56 studies of these met all the inclusion criteria and were therefore included in the review.

Overall, 99 articles were excluded. The main reasons for excluding documents during full texts screening included: a) studies did not relate to, or focus on, men; b) results or findings were not disaggregated by sex, c) articles were not focused on health literacy or health seeking behaviour.

### Bibliometric analysis and trends

#### Geographic distribution

Table 1 shows the distribution of studies across SSA sub-regions and countries. Of the 47 countries in the SSA region, 14 countries (30%) hosted all of the 56 studies retrieved. Three-quarters of all studies took place in East Africa (5 countries hosting 22 studies [30–51]) and in Southern Africa (4 countries hosting 21 studies [52–72]). The remaining quarter of studies took place in 3 West African countries [73–82] and 2 Central African countries [83–85].

**Table 1:** Distribution of studies by SSA regions and countries

Region	Countries (n)	Total studies
**East Africa** (5 countries)	Tanzania (*n* = 7), Uganda (*n* = 7), Kenya (*n* = 4), Ethiopia (*n* = 3), Rwanda (*n* = 1)	22
**Southern Africa** (4 countries)	Malawi (*n* = 8), South Africa (*n* = 5), Swaziland (*n* = 4), Zimbabwe (*n* = 4)	21
**West Africa** (3 countries)	Nigeria (*n* = 6), Ghana (*n* = 3), Senegal (*n* = 1)	10
**Central Africa** (2 Country)	DR Congo (*n* = 1), Cameroon (*n* = 2)	3
**TOTAL**	**14 countries**	**56**

#### Time-related distribution

**Fig. 2:**
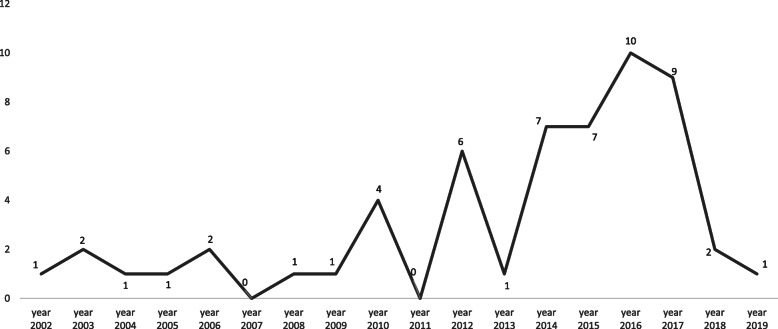
Yearly frequency of publication in men’s HL and SB in SSA since 2002

From 2000 to 2009, studies on men’s health literacy and health-seeking behaviour in SSA were almost non-existent with an average of one publication per year for the whole region. Since 2010, there has been a very small but steady increase in the number of publications, with 2016 being the most prolific year, recording a total of 10 publications for that year (see Fig. 2).

#### Study focus

HIV and SRH related topics were found to dominate the research agenda with 46 out of 56 (82%) included papers focusing either on SRH (*n* = 24; 43%) or on HIV-related issues (*n* = 22; 39%). Tuberculosis, male cancers, and general health issues accounted for 18% (*n* = 10) of the retrieved papers (see Table 2).

**Table 2:** Distribution of study focus for 56 included papers

Clinical area	Frequency of studies	References
Family planning and maternal and childcare	24	[30, 31, 35, 38, 39, 45, 47–51, 59, 62, 66, 67, 70–72, 74–76, 78, 80, 83]
HIV and STI related services, including HIV/TB.	22	[32–34, 37, 40, 43, 46, 52, 53, 56–58, 60, 61, 63, 64, 68, 77, 79, 81, 85]
Tuberculosis	4	[42, 44, 54, 55]
Non-communicable diseases (including cancers)	3	[65, 73, 82]
General health	3	[41, 69, 84]

#### Study methodology and design

There was a roughly equal balance between the proportion of studies that used qualitative (*n* = 26; 46%) and those using quantitative designs (*n* = 24; 43%). Mixed method studies constituted the minority (*n* = 6; 11%). Studies that used quantitative designs included: 17 cross-sectional surveys, five quasi-experimental designs (equivalent and non-equivalent group pre and post-test surveys), one post intervention survey and one unmatched case control study.

There were 20 studies employing qualitative designs: five of these used focus group discussions as the only data collection method, whereas 15 others combined focus groups with other qualitative methods, principally interviews. Five other studies used in-depth interviews (IDI) alone and one study combined IDI with participatory workshops for data collection.

#### Study population

Just over half of the studies included male participants only (*n* = 29; 52%). All studies related to males aged between 17 to 79 years. However, some comparative studies included female participants (*n* = 21; 38%). Of the 56 studies, six papers focused on men who have sex with men [MSM] and one study [46] involved male sex workers. Most participants were described as local community members, often from a low economic background. A few studies involved symptomatic patients such as men with a chronic cough, TB, sexually transmitted illnesses, or infertility. In six studies, men’s health in general was discussed but numbers and characteristics of male participants were not detailed.

#### Aim and scope of studies

We distinguished three types of study aims: descriptive, evaluative, and analytic. Descriptive studies (*n* = 28; 50%) aimed to provide a narrative account of men’s experiences of intervention, programmes and health services. Most studies in this group adopted a qualitative design due to the exploratory nature of their objectives. For example, many studies in this group explored the way masculinity and men’s specific knowledge, attitudes or perceptions in relation to diverse health issues including TB, HIV and family planning, influenced their engagement with specific health services [33, 35, 40, 52, 54, 77]. Other studies aimed to identify facilitators and barriers to men’s engagement with health programmes [36, 41, 85] and described men’s health practices and utilisation of health services [48, 60, 71]. Additionally, a few studies in this category provided details on men’s care pathways for specific diseases including symptoms recognition, decision making process, choice of and access to care [65, 66, 86].

Evaluation studies (*n* = 10; 18%) aimed to measure the impact of specific interventions on men’ s utilisation of health services or on men’s knowledge and attitudes towards health issues. For example, one of the papers assessed the impact of a community-based peer support group in maintaining participants’ engagement with antiretroviral therapy (ART) services and in preventing HIV [43].

In turn, analytic studies (*n* = 18; 32%) aimed to establish associations between masculinity norms and other social determinants of men’s health/male health behaviours and men’s utilisation of health services. Most studies in this group adopted a quantitative study design and used cross-sectional surveys. For instance, one study in this group used a cross-sectional survey to assess the association between social capital and HIV testing behaviour among MSM in Swaziland [57]. Another example studied the association of specific demographic variables—such as age, education and marital status— with risky sexual behaviours among adult heterosexual men in South Africa [64].

### Types of services and interventions studied

Depending on their aims, the 56 papers included can be classified into two broad types. First, about two-thirds of the studies (*n* = 39; 70%) reported on research describing men’s uptake, engagement, and or experience of routine health services offered and endorsed by national health systems. Most of these studies aimed to explore the low uptake of men in national programmes promoting uptake of antiretroviral therapy (ART), HIV- testing, TB, and family planning [34, 39, 46, 55, 74]. Many of these health services were delivered at facility level by health care workers.

Second, roughly a third of the studies (*n* = 17; 30%) reported on interventions or programmes introduced ‘de novo’ by external organisations, for example, donor agencies or non-governmental organisations, working in partnership with national health systems as a response to the observed low engagement of men in routine programmes and services. Examples of these studies included assessments of interventions to increase awareness and knowledge about specific health topics, to promote uptake of HIV testing, to improve screening for TB or to enhance men’s involvement in women’s health [33, 44, 56]. Most of these interventions were community-based and delivered using different mechanisms, for example, mass media, community health workers, or community gatherings or workshops (see Table 3).

**Table 3:** Intervention characteristics and outcomes

Intervention category	Intervention aims	Intervention activities	Intervention outcomes	Findings specific to men	Intervention gender typology	References
**Health campaigns and community mobilisation and sensitization interventions (*n*** **= 11)**	Aimed to create awareness, improve knowledge and attitude (through group education or mass media) on specific social and health issues—such as HIV related care and prevention [32] and gender-based violence [GBV] [30]—or provide specific health services or to encourage men and community members to utilise them	They involved activities such as diffusion of HIV information, promotion of condom uses and HIV testing, promotion of men’s involvement in MCH and promotion of gender equitable attitudes. For example, one such intervention involved community-based health education sessions about family planning using flyers, booklets, and group discussions (sometimes at household level) to promote husband-wife discussions on family planning [FP] and increase uptake of FP services [49].	It appeared that exposure to health campaigns resulted in improved health seeking behaviour, condom use and uptake of HIV tests [32, 33] especially when promoted health services were free of charges [53, 58]. Additionally, most media campaigns improved knowledge of and attitudes towards GBV, family planning, maternal health issues and HIV [30, 49, 61].	Particularly, men were reported to be interested in practical programmes such as those demonstrating proper use of condoms [32]. However, it was also noted that men’s gendered role of household’s breadwinner was one of the main barriers for men not attending health campaigns as they will often be away working [33]. Therefore, a need for a more inclusive approach, as regard to men, was identified, which would integrate specific men’s need for health education and address structural barriers of access to health information [32, 49].	Majority of interventions were either gender neutral (*n* = 4) [32, 53, 55, 58] or gender sensitive (*n* = 4). Among the later, two were urology related [59, 77], one involved MSM [48] and one intervention specifically introduced a form of monetary incentives for men [33]. Only one intervention was gender transformative [30]	[30, 32, 33, 48, 49, 53, 55, 58, 59, 61, 77]
**Community-based health services (*n*** **= 8)**		Health services provided included community-based TB diagnosis, HIV testing, care, and treatment and comprehensive SRH. Few other interventions were church based [73, 84]	Gender norms of traditional masculinity (bodily resilience, self-reliance, and control) and the perceived stigma relating to specific health conditions such as HIV or TB were described as main intersecting barriers for men to seek help for their health. It also appeared that locating health service facilities in places where men socialise, or where women and children frequent, amplified not only men’s anticipated stigma relating to illness itself but also men’s perceived threat to their adequate masculinity enactment [43]. However, the integration of income-generating activities in health programmes appeared to cushion the perceived stigma related to health seeking [43].	findings suggested that to improve men’s engagement with the community service delivery, there was need to implement interventions which integrate gender transformative and stigma-reduction dimensions [43, 47, 79]. it also appeared that specific age-group’s health needs for men have an important role when designing men health programmes [79].	Majority (*n* = 5) were gender-neutral [43, 44, 67, 73, 84] but two interventions were gender sensitive as they seemed to recognise and explore men specific health needs. In fact, one of these involved MSM [79]. One intervention was gender partnering [47]	[43, 44, 47, 63, 67, 73, 79, 84]
**Home-based training and health services** **(*n*** **= 1)**	Aimed to improve men’s and households’ members skills to recognize danger signs in pregnancy and promote health seeking behaviour and, to improve knowledge of attitude toward and men utilisation of contraceptives	consisted of a maternal and child health training conducted by a community health worker at household level, involving household’s members, including men.	The study found a significant improvement in male involvement and knowledge of maternal and child health issues. The proportion of men in the intervention group accompanying their wives to antenatal and delivery significantly increased as well as the frequency of shared decision-making for health matters.	The intervention showed a significant improvement in male involvement in women’s health and in the knowledge of danger signs during pregnancy, childbirth, and postpartum periods.	Gender partnering	[31]
**peer education** **(*n*** **= 1)**	Aimed to promote equitable gender, to transform harmful gender attitudes and behaviours, and to improve men’s engagement in FP and HIV services.	Use of male peer educator to act as peer models for groups of men and train other men during supervised community.	In relation to men’s health seeking, the intervention managed to achieve an increase in reported health-seeking behaviours such as visiting health facilities for health matters, using of condom with main partners, testing for HIV, and communicating with main partners on using a method to avoid pregnancy.		Gender transformative	[37]
**Health self-service** **(*n*** **= 1)**	Provision of self-test kits for HIV to men, delivered by women attending PMTCT clinics.	Aimed to improve male involvement in PMTCT	The approach was reported as widely accepted by men who, seemingly, expressed higher preference for it as compared to the standard facility-based testing, partly due to flexibility. Most men first preference was self-test alone, followed by testing as a couple	However, it was noted that post-test linkage remained an issue. Participants suggested that financial incentives and phone call reminders could be one way of addressing this.	Gender partnering	[56]

### Findings of the narrative synthesis

In line with our research questions, we first outline findings relating to how men are conceptualized across the reviewed literature and then explore how these conceptualizations relate to gender-responsiveness in services and interventions.

### Conceptualizations of men

Across interventions, programmes, or services depicted in the reviewed studies, we distinguished four principal ways in which men were conceptualized.

*Man, the gatekeeper (n = 22; 39%)* In these studies, men’ s socio-cultural positionality within the household and the community was emphasized. Men were described as the main decision-makers at household level, and therefore pivotal gatekeepers for health-related issues. Their social status and power must be harnessed for successful implementation of programmes targeting other population groups (women and children). This conceptualisation was most common in articles studying family planning programmes, for example, where women, who were the main recipient of care, needed consent from their male partner to engage with care [49–51, 75, 76, 85]. For example, a study from Nigeria notes that:

Nigerian men are often the final decision-makers on key household issues, including those related to household purchases, health of family members, timing of pregnancies, family size, and education of children … women-only programs or those that involve men in a limited way are not sufficient to bring about the magnitude of change in contraceptive use that is required for fertility decline at a national level ( [75] pE42)

*Masculine Man (n = 15; 27%)* In these studies, men and their health-related behaviours were described within a framework of the enactment of masculine norms and prescriptions. Here, men were often described as conforming to unhealthy behaviours related to cultural norms around masculinity, for example, as reluctant to seek care when sick, prone to risky sexual behaviour, participants to, and victims of, stigmatising attitudes, and having low levels of health-related knowledge (often tainted with misconceptions) [30, 32, 34, 52, 55, 58, 64, 68, 74]. For example, one article studying determinants of HIV-testing among men, noted that: “Men in Tanzania and Nigeria seek extramarital sexual partners to increase their sense of masculinity and self-esteem when faced with situations, such as unemployment …” ( [34] p.451)

In another study, conducted in Malawi, the authors elaborated on men’s health behaviours as directly related to cultural expectations of masculinity, arguing that: “Men’s pressures arising from failure to meet dominant expectations trigger a crisis that drives risk-taking and poor health behaviour.” ( [55] p2)

*Marginal Man, in a paradox (n = 9; 16%)* This conceptualisation described men as occupying a paradoxical or ambiguous position: their engagement was recognised as key for programmes’ success and disease control, but because of structural barriers limiting their access to, and uptake of services, they were often overlooked in program and health system design. This conceptualisation was predominant among papers that studied minority groups of men such as MSM [41, 48, 57, 69, 79]. Studying the role of gender in men’s health seeking for HIV and TB, Chikovore and colleagues noted, for example: “Despite men’s key role in TB transmission dynamics, relatively limited emphasis has been put on their epidemiological or social positions. When a gender perspective is incorporated into policy or research, the focus is often on women.” ( [54] p2)

*Man, the client (n = 6; 11%)* This conceptualisation of men was pragmatic and focused on identifying and understanding men’s health needs rather than explaining the causes of men’s poor health. Here, men were described as having distinct needs, different from other population groups, which needed to be addressed in a specific manner [33, 59, 63, 65, 77, 82]. For example, two studies of prostate cancer screening emphasized men’s expressed knowledge needs and concerns as significant factors susceptible to enhance men’s uptake of screening programmes [65, 82].

For four studies (7%), the conceptualisation of men could not be determined [42, 44, 73, 86].

### Gender-responsiveness of services and interventions

**Fig. 3:**
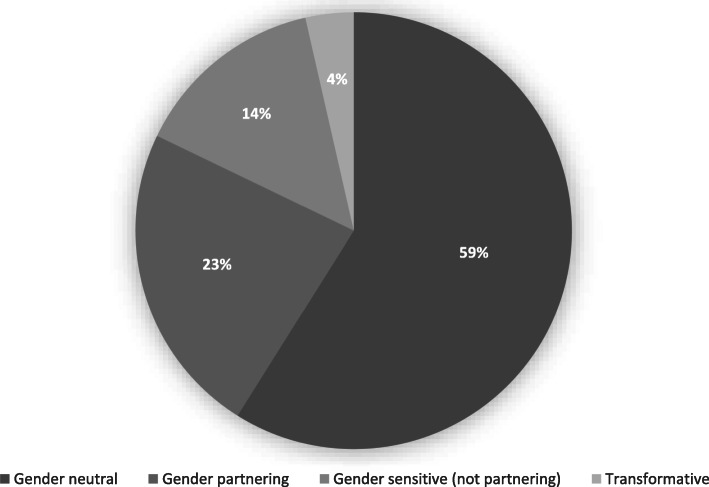
Gender responsiveness of interventions and services discussed across reviewed literature

Using the adapted version of the framework put forward by Gupta and colleagues [21], we grouped the included studies into four main categories (see Fig. 3 and Table 4).

**Table 4:** Gender responsiveness of the intervention and services discussed across reviewed literature

Intervention category	Category description	Example	References	Total nr. of studies
Gender neutral	Interventions/programmes which do not recognise gender differences in health needs and health seeking behaviour between men and women and provide undifferentiated health programming and services to men and women.	Studies in this category focused on most national TB and HIV programmes which provide a standardised ungendered health services to men and women [34, 39, 46, 53]	[32, 34, 35, 38, 39, 41–44, 46, 51–55, 57, 58, 60, 64–69, 71–73, 75, 78, 81, 83–85]	33
Partnering interventions	a form of gender sensitive programming which emphasises the key role of men in health issues of other population groups (women and children)	Most programmes in this group involve family planning programmes that aim to improve women uptake by finding ways to engage their male partners [40, 45, 76].	[31, 36, 40, 45, 47, 49, 50, 56, 61, 62, 70, 76, 80]	13
Gender sensitive	interventions/programmes which recognize differences in gender health needs and health seeking behaviour and where service delivery is adapted and implemented accordingly without an attempt to change gender norms within recipients	Such programmes have been delivered at facility level or as outreach interventions. In this review, this type of programming has mainly included those that addressed urologic health issues which are intrinsically masculine by nature including circumcision and prostate cancer screening (59, 74, 77, 82	[33, 48, 59, 63, 74, 77, 79, 82]	8
Gender transformative	interventions where the differences between men and women health needs and health seeking behaviour are recognised, and an attempt is undertaken to change, transform gender norms that are identified as unhealthy for men and women. Here emphasis is put on changing gender norms	interventions have included peer education using model men—male members of the community who have adopted progressive gender norms, to serve as role model [37]	[30, 37]	2

Thirty-three studies (59%) described services or interventions that could be classified as gender neutral. With the exception of one study describing a mass-media campaign for sexually transmitted infection (STI) prevention [32], all of these studies reported on men’s uptake and experience of routine health services that do not respond to gender-specific needs of individuals, although they may be inherently ‘gendered’ because programmes implicitly targeted women. Nearly all of these studies used data to make the case for greater attention to men’s health. For example, a study investigating treatment-seeking behaviour for infertility in Rwanda concluded that history-taking and counselling for infertility at health centres must involve men [35]. Another study, reporting on men’s negative perceptions and experiences of STI clinics in South Africa, concluded that men required better access *to* “high-quality, non-judgmental sexual health care services.” ( [58] p87). Similarly, a study addressing the issue of how masculinity norms deter men with chronic cough from seeking health care in a timely fashion, argued for “targeted approaches that address men’s particular concerns, for example …. privacy, a semblance of control, and flexibility” ( [63] p8).

Thirteen studies (23%) described gender-partnering interventions, programs, or services, that sought to engage men with the aim of facilitating or improving women’s SRH behaviours and outcomes. For example, interviews conducted with health workers in Tanzania elicited strategies for encouraging men to engage in family planning services but found that most of these did not reach the intended audience [45]. A number of studies examined interventions that promoted men’s involvement in maternity care. For example, one study from Uganda reported on traditional birth attendants’ role in sensitizing men “about both traditional and biomedical maternal issues when they interact in the process of giving care to women” ( [50] p3). Another study from Uganda, however, drew on interviews with men to highlight the barriers to uptake of couple HIV testing in antenatal care, which men found both stigmatising and unappealing [43].

A few studies (*n* = 8; 14%) discussed men’s uptake of gender-sensitive but not partnering services or interventions. These focused predominantly on services which needed to be specific to male populations, such as urogenital services or male fertility. Studies in this group focused on prostate cancer screening [82], circumcision [59, 77] and vasectomy [74] services respectively. A few other studies focused specifically on services for men who have sex with men [48, 79].

Just two studies (4%) focused on gender-transformative programmes or interventions. These included an evaluation of a community-based program used to improve knowledge and attitudes toward sexual violence in Tanzania [30], and a study that evaluated a male engagement intervention to transform gender norms and improve family planning and service uptake in Uganda [37].

### Conceptualizations of men and their relation to intervention or service typology

Table 5 outlines how the previously identified conceptualizations of men relate to the type of intervention or service gender-responsiveness.

**Table 5:** Intervention typologies and findings based on men conceptualization

Conceptualization of men	Gender-responsiveness of intervention/service	N	References
*Man, the client (n = 6)* [33, 59, 63, 65, 77, 82]	Gender neutral	1	[65]
	Gender sensitive	5	[33, 59, 63, 77, 82]
	Gender partnering	0	
	Gender transformative	0	
*Masculine man (n = 15)* [30, 32, 34, 35, 43, 52, 55, 58, 60, 64, 66–68, 71, 74]	Gender neutral	13	[32, 34, 35, 43, 52, 55, 58, 60, 64, 66–68, 71]
	Gender sensitive	1	[74]
	Gender partnering	0	
	Gender transformative	1	[30]
*Man, the marginal (paradox) (n = 9)* [41, 46, 48, 53, 54, 57, 69, 79, 81]	Gender neutral	7	[41, 46, 53, 54, 57, 69, 81]
	Gender sensitive	2	[48, 79]
	Gender partnering	0	
	Gender transformative	0	
*Man, the gatekeeper (n = 22)* [31, 36–40, 45, 47, 49–51, 56, 61, 62, 70, 72, 75, 76, 80, 83–85]	Gender neutral	8	[38, 39, 51, 72, 75, 83–85]
	Gender sensitive	0	
	Gender partnering	13	[31, 36, 40, 45, 47, 49, 50, 56, 61, 62, 70, 76, 80]
	Gender transformative	1	[37]
Unclassified (*n* = 4) [42, 43, 73, 78]	Gender neutral	4	[42, 43, 73, 78]
	Gender sensitive	0	
	Gender partnering	0	
	Gender transformative	0	

Where men have been conceptualized as **masculine men**, interventions, programs, and services described were noted to be predominantly neutral. For example, the HIV Universal test-and-treat programme in South Africa aims to expand HIV treatment to prevent ongoing transmission. This intervention implies removing eligibility criteria for starting HIV treatment [87] but uses the conventional structures for HIV service delivery, which make no specific distinction between men and women. In his analysis of threats to the successful implementation of this intervention in South Africa, Chikovore and colleagues describe issues of stigma and masculinity constructs as they affect men’s health seeking:

Men’s longer delay in seeking healthcare, … higher likelihood of late HIV diagnosis, … and lower likelihood of remaining in care and higher odds of experiencing deteriorating health after HIV diagnosis … has been attributed partly to stigma and fear and, more broadly, the pressures that they face to conform to socially valued representations such as having strength, control, agency, and earning capacity, and being competitive and also capable material providers … ( [52] p2)

Where men are conceptualized as **gatekeepers**, the majority of studies describe interventions, services or programs which are gender-partnering. For example, in a study describing involvement of men in PMTCT for the purpose of retaining women in ART care during pregnancy and postpartum care, Yende and colleagues described men’s social power and influence on household care-seeking decisions. They noted that: “Men have social and economic roles within families that can influence decisions related to the health of the mother and child, participation of men in ANC and family health is one potential solution to improve PMTCT outcomes.” ( [70] p1)

### Factors associated with increased health literacy and health seeking

Across the body of reviewed studies, we note a number of factors reported by study authors to be associated with increased health literacy and health seeking.

First, in relation to the wider social determinants of health, authors in the selected studies identify adoption of progressive gender norms, higher age, higher education, being married and exposed to health campaigns as predictors of higher men’s knowledge scores, health seeking behaviour, and participation in services such as HIV testing, family planning, and fertility clinics [34, 64, 68, 75, 76, 86]. Although not often explored, studies also noted employment and higher income as correlated to higher utilisation of services [35, 81].

Second, studies noted that illness and its consequences (such as inability to work and physical dependence) were perceived as disruptions to social life. These elements were seen to compromise the enactment of ideal masculine roles (e.g., as ‘breadwinner’) and traits such as strength, control, and self-reliance [52–55, 63].

Third, study authors discussed the role of religion, health beliefs and the origin of illness. Misconceptions about the supernatural causes of diseases, coupled with the influence of some religious beliefs, were identified as important determinants of men’s care seeking, characterised by a preference for alternative medicine to formal health services [36, 42] in a few cases. Additionally, health services (such as circumcision) that were perceived to be congruent with religious beliefs, were noted to achieve universal adherence among believers (e.g., circumcision in Muslim communities of Nigeria) [67, 77].

Across the majority of studies, health system design and accessibility for men appeared to play a significant role in men’s health. Clinic settings were frequently found to be inadequate for male needs: they were staffed by female nurses, had restricted daytime opening hours, and were closed during weekends, for example. These issues were noted to reflect health systems’ lack of responsiveness to accommodate men. Several studies also noted that men felt culturally uncomfortable to discuss their sexuality with a female health care worker, in clinics mostly attended by women and children and during opening times that did not take their working schedules into account [33, 47, 52, 58, 60, 62, 63, 72].

Additionally, health care workers, in general, appeared to be ill-equipped to address men’s health issues (especially men of minority groups). This was attributed to health systems being designed around traditional constructs of gender. For instance, studying health seeking practices among and provision practices for MSM in Malawi, Wirtz and colleagues found that:

Providers suggested that limited data on the size of and burden of HIV among MSM in Malawi begets a perception among service providers that MSM do not exist … lack of data has resulted in fewer cues to action or appropriate information that would enable providers to learn about and address the sexual health needs of MSM in a rights-affirming way within health care settings*.* ( [69] p8)

This situation might explain, for example, the observation that social participation (defined as an operationalized measure for social capital) appeared to be the most important predictor of utilisation of health services among minority groups of men such as men who have sex with men (MSM), and male sex workers [40, 41, 46, 58, 60, 69, 85].

## Discussion

This is one of the few reviews focused specifically on the literature around men’s health and experiences of engaging with interventions, services or programs aiming to improve health literacy and health seeking behaviour in sub-Saharan Africa. The review documents the state of the literature and range of interventions, programs and services deployed in the hopes of securing this goal. We offer a summary of how men and their behaviour have been conceptualized within this body of literature. Reflections on how these conceptualizations relate to intervention, program and service design further enable us to identify implementation gaps.

The review identified 56 studies focused on men’s health literacy and health seeking in Sub-Saharan Africa. Overall, we note a modest uptake in publications on the topic following 2010, likely linked to the increased interest in gender and wider awareness of the need to investigate how social determinants of health shape health literacy and health seeking. In line with donor and programmatic interest, we note that studies focus predominantly on HIV and SRH issues. The majority of studies carried out to date are descriptive in nature, however they represent relatively equal proportions of qualitative and quantitative methods, broadly reflective of wider trends in relation to studies focused on appraising the relation of gender with health literacy and health seeking.

Our inductive analysis of how men are conceptualized across reviewed studies matches the broader focus on HIV and SRH identified above. Men are predominantly conceptualized as either ‘gatekeepers’ to improve women’s health or as ‘masculine men’, whose habits and ideation of masculinity infringe upon their health seeking or compromise their health status (e.g., as when promiscuous behaviour is associated with increased likelihood of acquiring HIV). In most studies, men are viewed as self-reliant individuals, as persons in power and enjoying positions of strength. In turn, illness is perceived to compromise this position by introducing vulnerabilities, health seeking thus first implies accepting that the masculine role has been compromised. Few studies describe the ‘marginal men’ who inhabit a paradoxical role where their engagement is simultaneously described as pivotal yet marginalised through systemic and structural barriers. A minority of studies acknowledge men as clients, whose needs are specific; these studies predominantly focus on the delivery of interventions specific to male health needs (e.g., urogenital or fertility related programs).

In line with these conceptualizations, it is not surprising that most of the interventions, services or programs identified in the literature can be classified as gender-neutral or partnering. Gender-neutral services as described in this review do not explicitly take gender-related differences in health needs and health seeking behaviour into account but favour one-size-fits-all type of programming. In contrast, gender-sensitive ‘partnering’ interventions reported on this this review acknowledge differences in male behaviour and may engage with specific aspects of this, however, tend to instrumentalise men as a channel to achieve improved women’s or family health outcomes.

Unsurprisingly, very few studies documents gender-transformative interventions, likely because these are the most difficult to bring about. The two studies identified within this category suggest that these interventions had some effects in mitigating harmful health effects of negative constructions of masculinity.

Furthermore, studies noted that the overall design of health systems appeared to drive the current limitations of health services in their responsiveness towards men’s needs. Studies revealed that health care workers appear to be ill- equipped to address men’s health issues, which constituted a major barrier for men’s health seeking. In line with wider literature [88–92], our review has identified the need to advocate for a transformation of the health system itself to be more inclusive and considerate of men’s needs.

There have been recent global calls for action to address men’s health, with an appeal for gender sensitive and transformative interventions, however, the latter are not only challenging to implement but potentially also theoretically problematic in their over-emphasis on gender as compared to other axes of intersectionality [93]. Challenges in implementation relate primarily to the limited capacity of many LMICs to engage in gender-related programming: public sectors have been chronically underfunded and tend to prioritise women’s health. In particular, transformation of gender norms, which by definition requires more intersectoral and in many cases even legal action, may therefore not be of immediate priority. Health systems in such settings may find it easier to start by implementing gender-sensitive approaches, for example, by ensuring service delivery during particular opening hours or in locations more easily accessible by men.

Adopting a comprehensive search and study selection process, our scoping review went beyond the requirements of scoping and charting the data to offer a narrative summary of emerging trends relating to conceptualizations of men, intervention design and effectiveness. We acknowledge several limitations. First, this review builds on the assumption that the state of research reflects two intertwined issues: men’s limited uptake and utilization of services on the one hand (a practical point) and the lack of global health attention to men’s health issues as reflected in the design of health services (a discursive point). The literature alone provides only a partial view of where research and practice are with regards to addressing men’s health and is relatively silent on how these two limitations interrelate. Further, we note that the operational intervention typology we used was difficult to apply to routine service delivery or programming. Historically, health systems have been set up to offer undifferentiated or non-gender sensitive care, therefore gender-neutral programming was in most cases the de-facto position of the health system. Additionally, although all included studies reported on implemented interventions or programmes, very few included an explicit account of their theories of change. For most interventions and programmes, pathways through which change was supposed to occur, remained unclear. Given the aims of this review, focused on conceptualizations of men and an appraisal of gender intervention typologies, we have not quality appraised included studies. Finally, we have not conducted study selection and data extraction in duplicate, though we have attempted to mitigate selection bias via random checking of study selection and data extraction.

## Conclusion

This scoping review illustrates the different ways men are conceptualized in health programming and how this relates to the design of services in sub-Saharan Africa. By examining the relationship between gender responsiveness of health programmes and existing conceptualisations of men, we demonstrate that the dominant discourse in gender programming as reflected in the literature reviewed, remains of men as self-reliant individuals, and as persons in positions of power and strength. Despite this apparent ‘privilege’, men’s specific health needs are often neglected, and men are not encouraged to engage directly with health services unless these are linked to, and concomitantly impact on women’s health.

This review did not uncover much progress on gender-transformative programming. We suggest that steps in this direction will involve both discursive and practical shifts. Given the interconnectedness of global health priorities, programming, and research, greater critical attention to *how we conduct research* as well as *how we design health systems* is required to balance increased visibility to men’s health issues with the promotion of gender equity in norms and power dynamics**.**

## Supplementary Information

**Additional file 1.** Search strategy.

**Additional file 2.** Data extraction framework.

**Additional file 3.** Eligibility criteria.

## Data Availability

The datasets used and/or analysed during the current study are available from the corresponding author on reasonable request.

## Abbreviations

ANC: Antenatal care
ART: Antiretroviral therapy
CINHAL: Cumulative index to nursing and allied health literature
HIV: Human immunodeficiency virus
IDI: In-depth interview
KI: Key informant
LMIC: Low- and middle-income countries
MSM: Men who have sex with men
NCD: Non-communicable diseases
PMTCT: Prevention of mother to child transmission of HIV
PRISMA: Preferred reporting items for systematic reviews and meta analysis
SRH: Sexual and reproductive health
SSA: Sub-Sahara Africa
STI: Sexually transmitted infection
TB: Tuberculosis
ToC: Theory of change
WHO: World Health Organisation
