# Supplementary material for: Changing men or changing health systems? A scoping review of interventions, services and programmes targeting men’s health in sub-Saharan Africa
Source: Int J Equity Health. 2021 Mar 31;20:87. doi: 10.1186/s12939-021-01428-z (PMC8011198; doi:10.1186/s12939-021-01428-z)
Supplement: Supplementary file 1 — Additional file 1. Search strategy. [file 12939_2021_1428_MOESM1_ESM.pdf]

Appendix 1: Example of a search conducted on Medline (via EBSCOhost) on 22/05/19

#	Query	Results
S19	S14 AND S15 AND S16 AND S17 Limiters - Date of Publication: 20000101-20190531; Age Related: Adolescent: 13-18 years, All Adult: 19+ years; Language: English, French, Portuguese	45
S18	S14 AND S15 AND S16 AND S17	57
S17	S10 OR S11 OR S12 OR S13	150,504
S16	S5 OR S6 OR S7 OR S8 OR S9	522,053
S15	S1 OR S2 OR S3 OR S4	2,379,219
S14	(MH "Men's Health") OR "men's health" OR (MH "Men")	6,678
S13	(MH "Information Seeking Behavior) OR "health* information seeking"	2,301
S12	(MH "Health Knowledge, Attitudes, Practice") OR "health* knowledge"	104,928
S11	(MH "Health Literacy") OR "health* literacy"	8,674
S10	(MH "Patient Acceptance of Health Care") OR "health* seeking"	44,131
S9	Angola OR Benin OR Botswana OR "Burkina Faso" OR Burundi OR "Cabo Verde" OR Cameroon OR "Central African Republic" OR Chad OR Congo OR "Cote d'Ivoire" OR "Ivory Coast" OR Eritrea OR Ethiopia OR Gabon OR Gambia OR Ghana OR Guinea OR Kenya OR Lesotho OR Liberia OR Madagascar OR Malawi OR Mali OR Mauritania OR Mauritius OR Mozambique OR Namibia OR Niger OR Nigeria OR Rwanda OR "Sao Tome and Principe" OR Senegal OR Seychelles OR "Sierra Leone" OR Somalia OR "South Africa" OR Sudan OR Swaziland OR Eswatini OR Tanzania OR Togo OR Uganda OR Zambia OR Zimbabwe	502,022
S8	subSahara* OR sub-Sahara* OR "sub Sahara*" OR "South of the Sahara"	31,008
S7	"South Africa*" OR "South of Africa" OR "Southern Africa*"	107,091
S6	"East Africa*" OR "East of Africa" OR "Eastern Africa*"	10,895
S5	"West Africa*" OR "West of Africa" OR "Western Africa*"	15,505
S4	"health service*"	480,058
S3	(MH "Government Programs") OR (MH "National Health Programs") OR "program*"	1,269,396
S2	"campaign"	28,057
S1	"intervention*"	907,635
