# Supplementary material for: Changing men or changing health systems? A scoping review of interventions, services and programmes targeting men’s health in sub-Saharan Africa
Source: Int J Equity Health. 2021 Mar 31;20:87. doi: 10.1186/s12939-021-01428-z (PMC8011198; doi:10.1186/s12939-021-01428-z)
Supplement: Supplementary file 2 — Additional file 2. Data extraction framework. [file 12939_2021_1428_MOESM2_ESM.pdf]

Appendix 3 Data extraction framework

Main category	Subcategory	Description
**1. Author**		Last name, first name
**2. Year**		Year study conducted
**3. Country**		
**4. Title**		
**5. Objectives**		Stated objectives of the study
**6. Study method classification**	a. Qualitative<br>b. Quantitative<br>c. Mixed	
**7. Study design**		Describe study's methodology and methods in detail.
**8. Study population**		Number of participants and describe population group under study.
**9. Description of men**		Extract quotes/ describe how men and their roles are conceptualized in study
**10. Intervention – summary**		Summarize intervention
**11. Intervention – mode of delivery**		Settings, implementers, and modalities of implementation
**12. Intervention – length / intensity**		Time frame and frequency of implementation/ exposure
**13. Intervention – TOC**		Summarize theory of change of intervention, if available
**14. Intervention TOC quote**		Extract any quotes on intervention theory of change here
**15. Outcome variables**		Outcome measures
**16. Main Findings**		Main findings with respect to men
**17. Notes**		
