# Supplementary material for: Changing men or changing health systems? A scoping review of interventions, services and programmes targeting men’s health in sub-Saharan Africa
Source: Int J Equity Health. 2021 Mar 31;20:87. doi: 10.1186/s12939-021-01428-z (PMC8011198; doi:10.1186/s12939-021-01428-z)
Supplement: Supplementary file 3 — Additional file 3. Eligibility criteria. [file 12939_2021_1428_MOESM3_ESM.pdf]

Appendix 2: Eligibility criteria

Domain	Criteria
**Time restriction**	Study published since 2000 (motivated by the desire to focus on recent evidence in this field)
**Language restriction**	Study published in English, French, Portuguese.
**Population**	Study focuses on men; at least 15 years old; of any sexual orientation.
**Intervention**	Study reports on services, programmes, and interventions
	Study reports a theory of change and/or offers an account of intervention implementation and results.
**Outcomes or topical focus**	Study outcomes or study focus include health literacy or engagement with health services or health seeking.
**Country setting**	Study is conducted in one of the 47 countries of the Sub-Saharan Africa region.
**Study type**	Study is a qualitative study of any type; or a quantitative study (including controlled before and after, interrupted time series or trials).
